# [Corrigendum] Lysyl oxidase-like-1 enhances lung metastasis when lactate accumulation and monocarboxylate transporter expression are involved

**DOI:** 10.3892/ol.2026.15867

**Published:** 2026-09-16

**Authors:** Geum-Hwa Lee, Do-Sung Kim, Myung Ja Chung, Soo-Wan Chae, Hyung-Ryong Kim, Han-Jung Chae

Oncol Lett 2: 831–838, 2011; DOI: 10.3892/ol.2011.353

Subsequently to the publication of the above article, an interested reader drew to the authors' attention that, concerning the cellular invasion assay data shown in Fig. 3A on p. 835, data shown in the ‘LOCL-1 siRNA’ panel were strikingly similar to data that had already appeared in an article written by the same research group, which was published in the journal *Oncogene*.

After re-examining their original data, the authors were able to confirm that Fig. 3 had been assembled incorrectly in the above paper. A revised version of Fig. 3, now showing the correct data for the ‘LOCL-1 siRNA’ panel in Fig. 3A, is shown on the next page. Note that the revision made to this figure has not led to any significant changes in either the results or the conclusions reported in this paper. The authors are grateful to the Editor of *Oncology Letters* for allowing them this opportunity to publish a Corrigendum, and all the authors agree with its publication; furthermore, they apologize to the readership for any inconvenience caused.

## Figures and Tables

**Figure 3.**
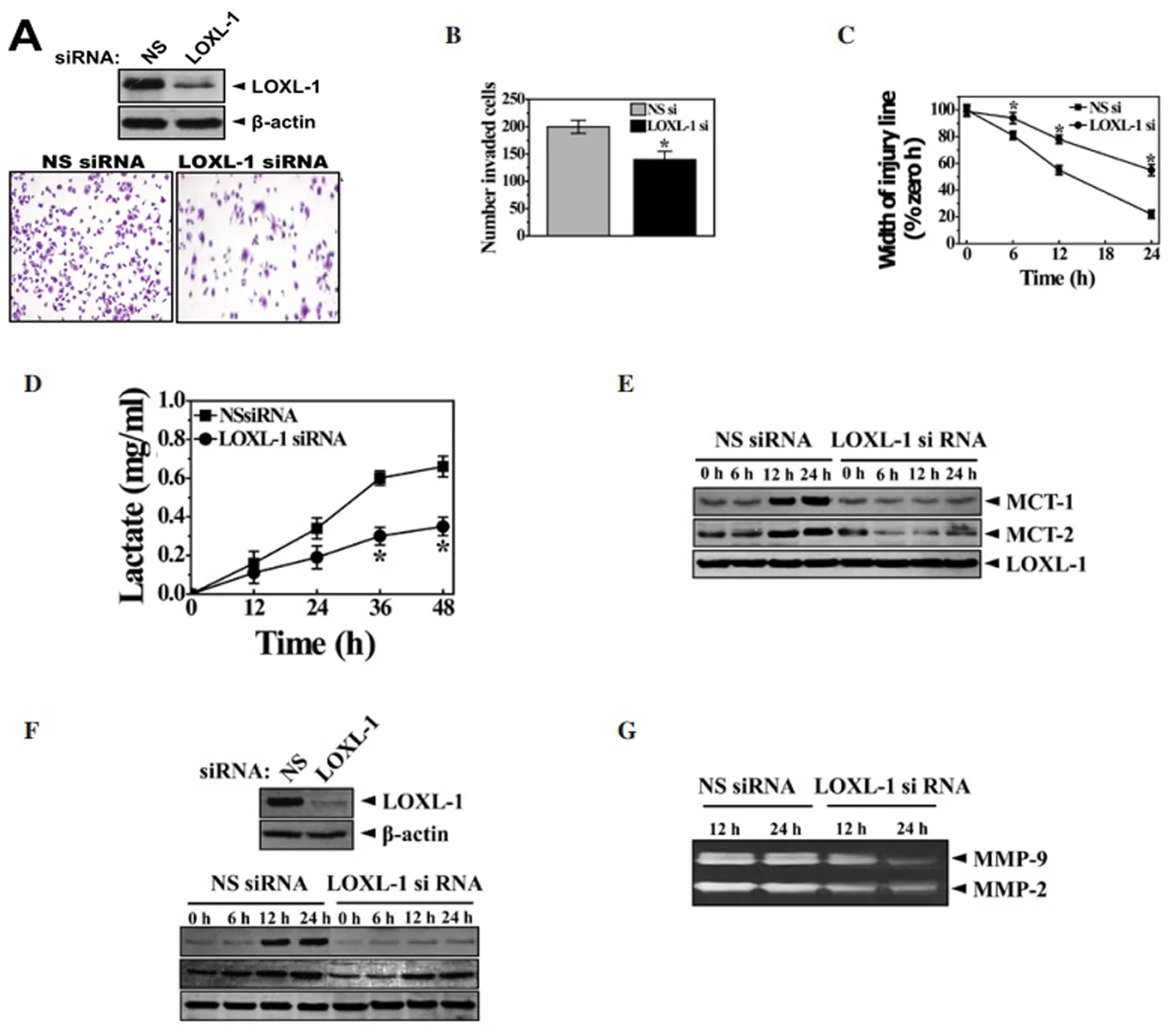
Endogenous LOXL-1 plays a significant role in the extracellular accumulation of lactate and the expression of its transporter, MCT1/2, in *in vitro* cancer metastasis. (A) HT1080 cells were transfected with non-specific or LOXL-1-specific siRNA and the expression of LOXL-1 was confirmed (upper panel). The invasion analysis was performed as in Fig. 2A. The 0.1% crystal violet staining cells in the lower chamber are shown (A, lower panel). (B) The number was determined by counting cells in the lower wells (C). The width of the injury line was quantified at the indicated culturing periods, 0, 6, 12, 18 or 24 h. (D) Lactate in the medium was measured when non-specific and LOXL-1 siRNA-transfected cells were cultured for the indicated periods (12, 24, 36 or 48 h) (E and F) Western blotting was performed with anti-MCT1/2, MMP2/9, LOXL-1 or β-actin antibodies. (G) Zymography was performed for MMP2/9 activity. *P<0.05, significantly different from the non-specific siRNA-transfected condition. NSsi, non-specific siRNA.

